# The effects of heavy resistance training and a high protein diet (3.4g/kg/d) on body composition, exercise performance and indices of health in resistance-trained individuals - a follow-up investigation

**DOI:** 10.1186/1550-2783-12-S1-P37

**Published:** 2015-09-21

**Authors:** Anya Ellerbroek, Corey A Peacock, Steve Orris, Max Scheiner, Adriana Gonzalez, Tobin Silver, Jose Antonio

**Affiliations:** 1 Exercise and Sports Sciences, Nova Southeastern University, 3532 S. University Drive, University Park Plaza Suite 3532, Davie FL 33314, USA

## Background

The consumption of a high protein diet (> 4g/kg/d) in trained men and women who did not alter their training program has been previously shown to have no significant effect on body composition. Thus, the purpose of this investigation was to determine if a high protein diet in conjunction with a body part, split-routine heavy resistance training program would affect indices of body composition, performance and health.

## Methods

Forty-eight healthy resistance-trained men and women completed this study (mean ± SD; Normal Protein group [NP n = 17 four female and 13 male]: 24.8 ± 6.9 yr; 174.0 ± 9.5 cm height; 74.7 ± 9.6 kg body weight; 2.4 ± 1.7 yr of training. High Protein group [HP n = 31 seven female and 24 male]: 22.9 ± 3.1 yr; 172.3 ± 7.7 cm; 74.3 ± 12.4 kg; 4.9 ± 4.1 yr of training). Subjects in the NP and HP groups consumed 2.3 and 3.4g/kg/day of dietary protein during the treatment period. Moreover, all subjects participated in a split-routine, body part heavy resistance-training program. Training and diet (everyday) logs were kept by each subject.

## Results

A two-time point (Pre, Post) by two-group (NP, HP) repeated-measures analysis of variance (ANOVA) was utilized to examine body composition measures. There were significant time by group (p ≤ 0.05) changes in body weight (1.3 ± 1.3 kg NP, -0.7 ± 4.0 HP), fat mass (-0.3 ± 2.2 kg NP, -1.7 ± 2.3 HP), and % BF (-0.7 ± 2.8 NP, -2.4 ± 2.9 HP) in the HP group. There was a significant time effect for FFM for both groups; however, the time by group effect FFM (1.5 ± 1.8 NP, 1.5 ± 2.2 HP) was not significant. Furthermore, a significant time effect (p ≤ 0.05) was seen in both groups vis a vis improvements in maximal strength (i.e., 1-RM squat and bench) vertical jump and pull-ups; however, there were no significant time by group effects (p ≥ 0.05) for all exercise performance measures. Additionally, there were no changes in any health parameters (i.e., basic metabolic panel).

## Conclusion

Consuming a very high protein diet (3.4g per kg daily) in conjunction with a heavy resistance-training program may confer benefits with regards to body composition. Furthermore, there is no evidence that consuming a high protein diet causes any adverse effects.

